# Influence of carbohydrate polymer shaping on organic dye adsorption by a metal–organic framework in water†

**DOI:** 10.1039/d1ra03348d

**Published:** 2021-07-06

**Authors:** Yutaro Tanimoto, Shin-ichiro Noro

**Affiliations:** Graduate School of Environmental Science, Hokkaido University Sapporo 060-0810 Japan; Faculty of Environmental Earth Science, Hokkaido University Sapporo 060-0810 Japan noro@ees.hokudai.ac.jp

## Abstract

A number of studies have been conducted to develop new metal–organic frameworks (MOFs) as adsorbents for the removal of contaminants from polluted water. However, few reports exist describing detailed and thorough examinations of the effects of shaping on the adsorption properties of MOFs. In this study, a thorough analysis and comparison was conducted of the Orange II and Rhodamine B dye adsorption properties of unshaped MIL-100(Fe) (MIL) particles and alginate polymer-shaped MIL beads (MIL-alg). The adsorption affinities of Orange II and Rhodamine B for unshaped MIL were observed to be higher than those for shaped MIL-alg because partial coating of the surface of MIL particles by alginate polymer weakens adsorption forces. Kinetic analysis using a two-compartment model indicates that the contribution of the slow step in the mechanistic pathway for adsorption is more pronounced in MIL-alg compared to MIL because slow dye diffusion takes place in the alginate polymer. We believe that these fundamental findings will have a beneficial impact on approaches to design shaped MOFs that display improved dye removal performance.

## Abstract 2

A thorough analysis and comparison was conducted of the Orange II and Rhodamine B dye adsorption properties of unshaped MIL-100(Fe) (MIL) particles and alginate polymer-shaped MIL beads (MIL-alg).

## Graphical abstract

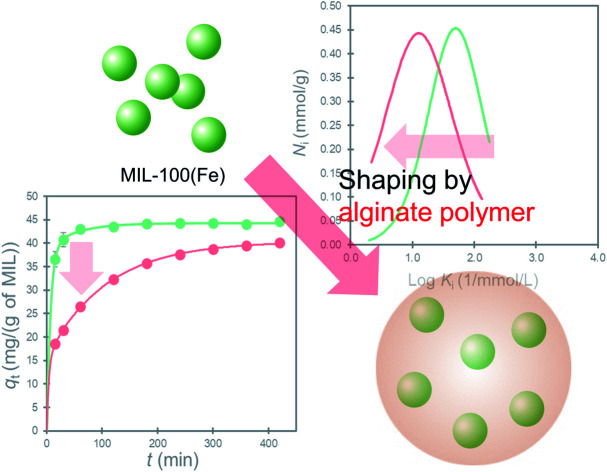

## Introduction

Because water is an indispensable substance for sustaining life, its pollution caused by several contaminants such as heavy metal ions, dyes and other organic compounds is a serious world-wide problem. Dye contaminated wastewater discharged in large quantities from textile manufacturing facilities is a major cause of water pollution. The presence of dyes in water is not only aesthetically unpleasant, but it also negatively impacts the aquatic environment. Various wastewater remediation methods have been devised thus far including those that rely on catalytic processes, ozonation, bioremediation and adsorption.^1,2^ Approaches which utilize adsorption have unique advantages associated with no external energy consumption, ease of operation and environmental compatibility. Therefore, many studies have been concentrated on developing strategies for dye removal from water, and those that utilize adsorbents such as activated carbons, zeolites and metal–organic frameworks (MOFs) have attracted much attention.^3–5^

MOFs are crystalline porous materials that consist of metal ions and organic ligands. Owing to their high surface areas and tunable pore properties, MOFs can be employed for gas separation and storage, and as chemical sensors and catalysts.^5–10^ Recently, the use of microcrystalline MOFs for removing pollutants such as heavy metal ions and dyes in aqueous media has been studied.^11–14^ To be compatible with practical applications, MOFs must be shaped into bigger agglomerates than microcrystals for easy handling. In this regard, MOFs have been shaped into hydrogels, fibers and membranes using appropriate diluents.^15,16^ It is important to recognize that diluents utilized to promote proper shaping can have a significant effect on the performance of MOFs such as dye adsorption rates, capacities and energies. However, most studies carried out thus far have focused only on the properties of shaped MOFs and only few efforts have been conducted to compare dye adsorption properties of shaped and unshaped MOFs.

**Fig. 1:**
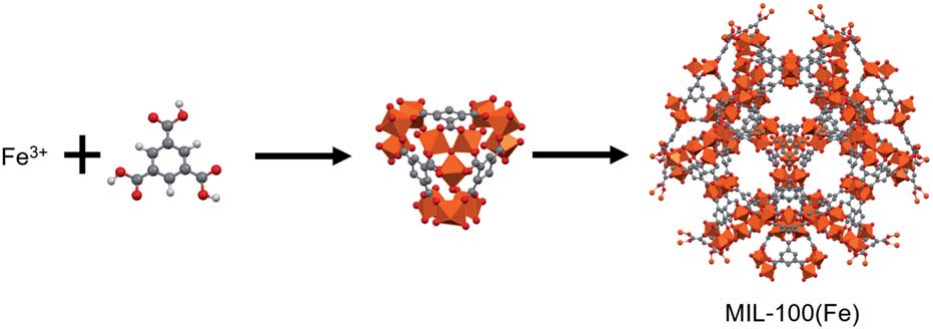
Structure of MIL-100(Fe).

MOFs that are appropriate for use in water remediation must be both green and sustainable in that the metal ion and organic ligand components must be non-toxic, readily available, and that the MOFs must be stable in water and easily synthesized using environmentally friendly conditions. Because it meets these prerequisites, the MOF, MIL-100(Fe) (denoted as MIL), constructed from trivalent Fe^3+^ cations and 1,3,5-benzenetricarboxylic acid ligands (Fig. 1),^17,18^ has been utilized as a potential adsorbent for removing organic and inorganic pollutants in aqueous solution.^12,19–23^ Fe is a very abundant less toxic metal, and 1,3,5-benzenetricarboxylic acid is commercially available and has been used for the synthesis of a variety of MOFs.^17,18,24,25^ Moreover, MIL is highly stable in water because it contains a strong Fe^3+^ trivalent cation–COO^−^ monoanion coordination bond. Furthermore, it was recently reported that this MOF can be synthesized in aqueous solution without using additives.^17^

**Fig. 2:**
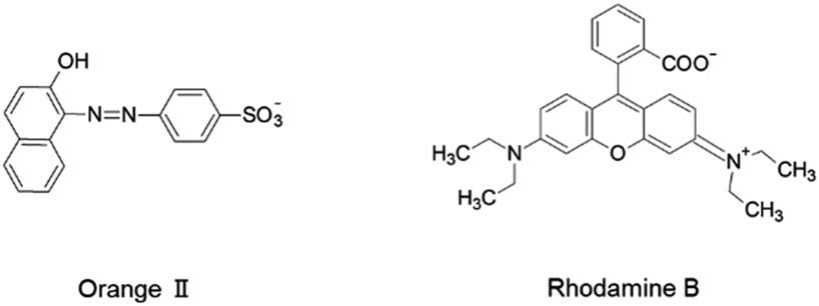
Molecular structures of dyes.

In the investigation described below, we thoroughly characterized and compared the organic dye adsorption properties of bead-shaped MIL particles, prepared using an alginate polymer, and their unshaped counterparts. Alginate polymer, a naturally occurring and environmentally friendly polysaccharide obtained from brown seaweed, is composed of 1,4*-*linked β-d-mannuronic (M block) and α-l-guluronic (G block) acid moieties. The presence of a multitude of carboxyl and hydroxyl groups enables the alginate polymer to cross-link *via* coordination to a variety of divalent and trivalent metal cations (Ca^2+^, Cu^2+^ and Fe^3+^) to form hydrogel beads. Therefore, the alginate polymer has been utilized as a shaping agent for a variety of adsorbents including MOFs.^26–31^ The dye adsorption properties of unshaped and bead-shaped MIL were investigated using Orange II and Rhodamine B as dyes, which have different chemical structures and charges (Fig. 2). Notably, the results show that shaping of MOFs not only affects adsorption affinities but it also impacts rates of dye adsorption.

## Experimental section

### Materials

All chemicals and solvents were purchased from FUJIFILM Wako Pure Chemical Corp. and used as received.

### Synthesis of MIL-100(Fe)

MIL-100(Fe) (MIL) was synthesized using a previously described procedure.^17^ A suspension of Fe(NO_3_)_3_·9H_2_O (4.04 g, 10.0 mmol) and 1,3,5-benzenetricarboxylic acid (1.89 g, 9.00 mmol) in water (6.0 mL) was stirred at 95 °C for 12 h. After heating, the solid was separated, washed with water and ethanol each three times at 70 °C for 24 h, and dried at 150 °C for 6 h. Elemental analysis of the solid showed that it is MIL having the molecular formula [Fe_3_O(H_2_O)_2_(OH)_0.7_(NO_3_)_0.3_(1,3,5-benzenetricarboxylate)_2_].

### Preparation of alginate polymer–MIL composite beads and alginate polymer beads

MIL-100(Fe) (500 mg) was dispersed in 100 mL water containing sodium alginate (300–400 cp, 2.0 g). The resulting suspension was vigorously stirred for 2 h and then dripped from a glass tube with inner diameter of 0.8–1.4 mm into an aqueous CaCl_2_ solution (0.1 M) at a constant rate using a peristaltic pump to form alginate polymer–MIL composite beads (denoted as MIL-alg). In order to complete the crosslink reaction, the beads were kept in the CaCl_2_ solution for 24 h. The beads were then washed three times with distilled water and stored in distilled water. The alginate polymer beads (denoted as alg) were prepared using the same procedure as that used to generate MIL-alg except that MIL was not included. The results of elemental analysis and energy dispersive X-ray fluorescence (XRF) spectroscopy showed that the formula of MIL-alg is (MIL)(alg)_4.92_(H_2_O)_14_.

### Characterization

Elemental analysis (carbon, hydrogen and nitrogen) was performed at the Global Facility Center, Hokkaido University, using a CE-440 elemental analyzer (Exeter Analytical, Inc.). X-ray photoelectron spectroscopy (XPS) measurements were performed using a JEOL JPS-9200 spectrometer with Al Kα radiation at 10 kV and 10 mA. XRF spectra were recorded using a Bruker S2 PUMA analyzer. Field-emission scanning electron microscopy (SEM) with energy dispersive X-ray spectroscopy (EDX) was conducted on a JEOL JSM-6510LA microscope at an accelerating voltage of 20 kV. Powder X-ray diffraction (PXRD) data were collected on a Bruker D2 PHASER 2nd Generation diffractometer with Cu Kα radiation (*λ* = 1.5418 Å) at room temperature. UV-vis absorption spectra were recorded on a JASCO V-770 spectrophotometer with a resolution of 4 cm^−1^. Attenuated-total-reflection infrared (ATR-IR) spectra were recorded using a Thermo Fisher Scientific Nicolet iS10 FT-IR spectrometer equipped with a GladiATRTM accessory with a resolution of 4 cm^−1^. Thermogravimetric analysis (TGA) was conducted using a TA instruments TGA Q50 and a RIGAKU TG-DTA 8122 in a 60 mL min^−1^ N_2_ flow at a heating rate of 10 °C min^−1^. N_2_ adsorption/desorption isotherms at 77 K were measured using a Microtracbel BELSORP-mini surface area and pore size distribution analyzer. Before adsorption/desorption experiments, samples were heated at 100 °C for 12 h under reduced pressure (<10 Pa) using a Microtracbel BELPREP VAC II.

The amount of iron leaked from MIL into water was determined using a phenanthroline colorimetric method as follows. MIL microcrystals (0.05 g) were immersed in 20 mL water for 24 h. The resulting suspension was centrifuged and the supernatant was removed. After adding 5.0 mL of supernatant and 20 mL of pure water to a 50 mL of volumetric flask, 10 mg of ascorbic acid was added to reduce Fe(iii) to Fe(ii). 1,10-Phenanthroline hydrochloride monohydrate solution (2.5 mL of 5.0 × 10^−2^ M) and 2.5 mL of buffer (pH 4.6), prepared from sodium acetic acid and glacial acetic acid, were added to the volumetric flask and the volume was adjusted to 50 mL. After 20 min, the absorbance of the solution at *λ*_max_ = 510 nm was measured using a UV-vis spectrophotometer.

### Dye adsorption experiments

Orange II and Rhodamine B were used to evaluate dye adsorption properties of MIL, MIL-alg and alg in aqueous solutions. All equilibrium and kinetic experiments were conducted in a batch process. In order to compare adsorption capacities of MIL itself and MIL in MIL-alg, the actual content of MIL in MIL-alg was determined using elemental analysis, XRF and TGA (Table S1 and Fig. S1, S2†). Equilibrium experiments were conducted as follows. 1.5 mg of MIL microcrystals, ten MIL-alg beads, which include 1.5 mg of MIL microcrystals, or ten alg beads were added to each dye solution (5 mL) containing different concentrations of Orange II (20–300 mg L^−1^) and Rhodamine B (20–120 mg L^−1^). The pHs of the Orange II and Rhodamine B dye solutions were 6.90 and 5.50, respectively. Each solution was then shaken at 100 rpm in a mechanical shaker at 30 °C for 24 h. After shaking, the supernatants were separated and their absorbances at *λ*_max_ = 485 nm for Orange II and *λ*_max_ = 552 nm for Rhodamine B were determined using a UV-vis spectrophotometer. The equilibrium adsorption amount (*q*_e_, mg (g of MIL)^−1^) at each dye concentration was calculated using eqn (1),1
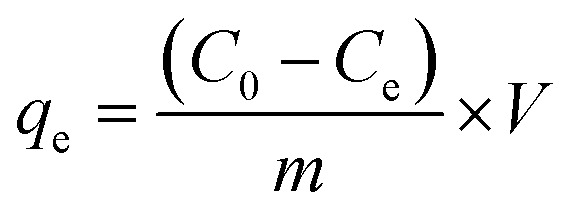
where *C*_0_ is the initial concentration of dye (mg L^−1^), *C*_e_ is the equilibrium concentration of dye (mg L^−1^), *V* is the solution volume (L) and *m* is a mass of dried MIL (g). Kinetic experiments were carried out using similar conditions except that the shaking time and dye concentration were 15–420 min and 15 mg L^−1^, respectively. The time-dependent adsorption amounts (*q*_*t*_, mg (g of MIL)^−1^) were calculated using eqn (2),2
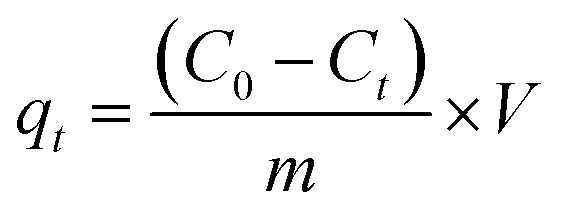
where *C*_*t*_ is the concentration of the dye (mg L^−1^) at each shaking time. The experiments were repeated three times and average values were plotted. Fitting of isotherms and kinetic analyses was performed using the nonlinear least squares method.

## Results and discussion

### Characterization of MIL, MIL-alg and alg

**Fig. 3:**
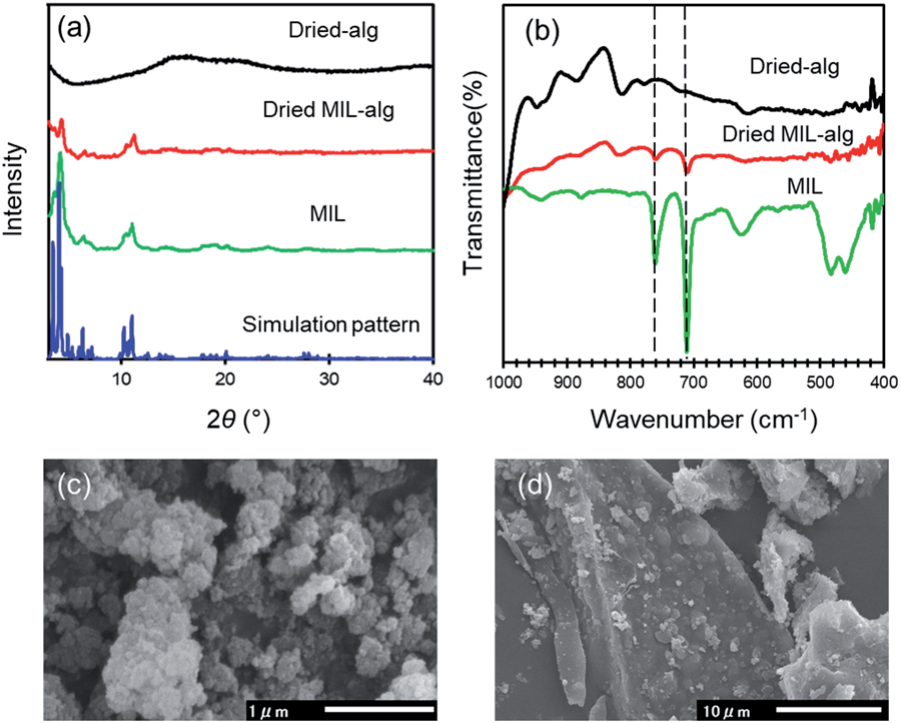
Characterization of MIL, dried MIL-alg and dried alg. (a) PXRD patterns of simulated and as-synthesized MIL, dried MIL-alg, and dried alg. (b) IR spectra of MIL, dried MIL-alg and dried alg. (c) and (d) SEM images of (c) MIL and (d) dried MIL-alg.

MIL, MIL-alg and alg were characterized using colorimetry, PXRD, SEM-EDX, ATR-IR, TGA, XRF, elemental analysis and N_2_ adsorption/desorption measurements. Generally, hydrogen fluoride (HF) is used as a crystallizing agent to obtain highly crystalline MIL.^19–22^ However, from the viewpoint of green chemistry, toxic HF was not used in the preparation of MIL employed in this study.^17^ The obtained MIL is stable in water, which was confirmed by the result of colorimetric measurements that show no leakage of iron ions from MIL into water. The positions of diffraction peaks in the PXRD pattern of MIL are similar to those in the simulated pattern calculated using crystallographic data (Fig. 3(a)). This finding indicates that a three-dimensional porous framework exists in this MOF.^32^ However, the diffraction peaks are very broad. The SEM image of MIL shows that it comprised of aggregates of small particles with size of ∼0.1 μm (Fig. 3(c)) that is slightly smaller than that of highly crystalline MIL-100(Fe) (∼0.6 μm) synthesized using same procedure.^17^ Therefore, the broadening of diffraction peaks is caused by low crystallinity. Differences between the detailed synthetic procedures, which was not described in original paper, may affect the crystallinity. The PXRD pattern of the dried MIL-alg beads contains a broad peak around 3–5° and 10–11° that is not observed in dried alg beads, suggesting that MIL is retained in the alginate polymer beads. The characteristic peaks at 710 and 762 cm^−1^, corresponding to C–H out-of-plane bending vibrations of the benzene ring, are present in the IR spectra of both MIL and dried MIL-alg but not in alg (Fig. 3(b)),^33^ again confirming the existence of MIL in the alginate polymer beads. To confirm the chemical composition of MIL-alg, elemental analysis and XRF were performed. The observed composition is MIL : alginate polymer = 1.0 : 9.84 (mol), a value that is close to the feed ratio (MIL : alginate polymer = 1.0 : 10.8 (mol)) (see Fig. S1 and Table S1†). The N_2_ adsorption/desorption isotherms of MIL are a typical type-I (Fig. S3†) and the BET surface area is calculated to be 1286 m^2^ g^−1^, smaller than that of highly crystalline MIL (1626 m^2^ g^−1^)^21^ and larger than that of nanoscale MIL (1.24 m^2^ g^−1^).^23^ SEM-EDX analysis of dried MIL-alg (Fig. 3(d), S6, S7 and Tables S4, S5†) indicates that MIL microcrystals are heterogeneously embedded in large alginate polymer agglomerations.

### Dye adsorption properties of MIL, MIL-alg and alg

**Fig. 4:**
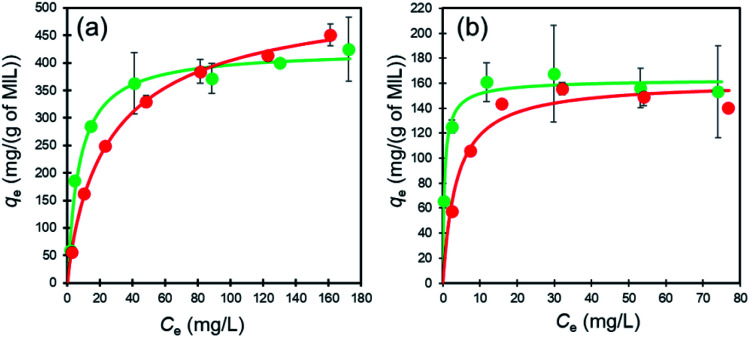
The adsorption isotherms of (a) Orange II in MIL (green circles) and MIL-alg (red circles) and (b) Rhodamine B in MIL (green circles) and MIL-alg (red circles) at 30 °C.

To examine adsorption of the dyes on MIL and MIL-alg in aqueous solutions, temporal changes in the absorption spectra of Orange II (*λ*_max_ = 485 nm) and Rhodamine B (*λ*_max_ = 552 nm) were monitored. As can be seen by viewing Fig. S8 and S9,† the intensities of absorption maxima for both dyes do not change in the presence of alg, whereas they decrease in a time dependent manner when MIL and MIL-alg are present. These results indicate that MIL serves as an adsorbent for Orange II and Rhodamine B. In Fig. 4 are shown adsorption isotherms for the dyes on MIL and MIL-alg. In low concentration regimes, the dyes adsorb on both MIL and MIL-alg. Fitting these isotherms using the Langmuir–Freundlich (LF) model (eqn (S1), see ESI†) affords *n* = 1.0 or close to 1.0 (Table 1), indicating that the adsorption behaviors are monolayer type and homogeneous adsorption sites are utilized.

**Table 1:** Langmuir–Freundlich fitting parameters for the dye adsorption isotherms of MIL and MIL-alg at 30 °C

	Adsorbent	*q*_m_ (mg (g of MIL)^−1^)	*a* (L mg^−1^)	*n*	*R* ^2^
Orange II	MIL	424	0.14	1.00	0.996
	MIL-alg	541	0.056	0.86	0.998
Rhodamine B	MIL	164	1.94	0.81	0.998
	MIL-alg	161	0.28	1.00	0.927

**Fig. 5:**
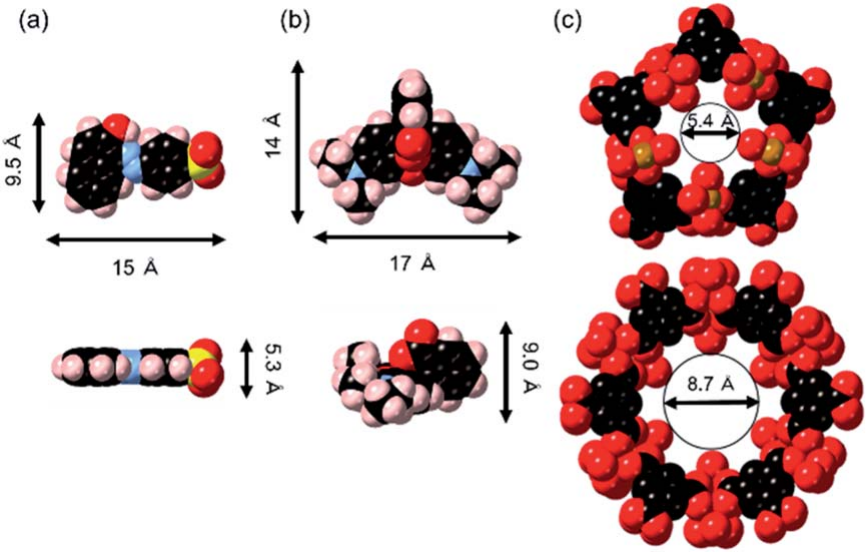
Molecular structures of (a) Orange II, (b) Rhodamine B and (c) two types of pore cage of MIL drawn by a space filling model. The vermilion, blue, black, red, pink and yellow colors represent iron, nitrogen, carbon, oxygen, hydrogen and sulfur, respectively.

The maximum amounts of Orange II adsorbed on MIL (424 mg (g of MIL)^−1^) and MIL-alg (541 mg (g of MIL)^−1^), obtained by fitting isotherms, are 2.6 and 3.4 times higher than those for Rhodamine B adsorption (164 mg (g of MIL)^−1^ and 161 mg (g of MIL)^−1^ for MIL and MIL-alg, respectively). In Table S6† are listed the adsorption amounts of Orange II and Rhodamine B on previously reported adsorbents. MIL and MIL-alg show moderate dye adsorption amounts among previously reported adsorbents. Tsai *et al.* reported that highly crystalline and porous MIL (BET surface area = 2037 m^2^ g^−1^), synthesized using HF, has a saturated Orange II adsorption amount of 410 mg (g of MIL)^−1^ at 25 °C,^22^ which is comparable with amount adsorbed by our less crystalline and less porous MIL. The saturated adsorption amount of Rhodamine B on MIL (164 mg (g of MIL)^−1^) is only about 2.4 times greater than the reported values obtained using nanoscale MIL-100(Fe) (69 mg (g of MIL)^−1^ at 25 °C and 73 mg (g of MIL)^−1^ at 35 °C) that has an extremely low BET surface area of 1.24 m^2^ g^−1^.^23^ These comparisons suggest that the amounts of the dyes adsorbed to MIL are not solely related to BET surface areas. In order to gain information about dye adsorption states, N_2_ adsorption/desorption isotherms of MIL containing saturating amounts of adsorbed dyes were measured (Fig. S4†). In the cases of both Orange II and Rhodamine B adsorbed MILs, N_2_ adsorption still takes place but the observed micropore volumes are smaller than those derived using the assumption that all dye molecules are adsorbed only on the outer surface of MIL (Table S2†). We also calculated the external surface area of MIL and theoretical monolayer adsorption amounts of Orange II and Rhodamine B on the external surface of MIL (see Table S3†). The theoretical values were considerably lower than the experimental values. These results suggest that the adsorption sites for both dyes in MIL are the insides of pores. Although the minimum molecular areas of Orange II (5.3 × 9.5 Å^2^) and Rhodamine B (9.0 × 14 Å^2^) are larger than the pore window sizes of MIL (5.4 Å and 8.7 Å) (Fig. 5), the porous framework is not static but dynamic in nature. For example, the replacement of building blocks (metal ions and organic ligands) occurs in a variety of MOFs including thermally stable Zr MOFs and zeolitic imidazolate frameworks with the aid of solvents.^34^ In addition, MIL-100(Fe) used in this study shows low crystallinity, which was confirmed by analysis of PXRD patterns, meaning that there are many defects in MIL frameworks. Considering these results, Orange II and Rhodamine B have the possibility of entering pores. The issue of reusability was evaluated for MIL-alg. Although the dye-adsorbed MIL-alg beads were immersed in solutions of water, ethanol, 0.1 M NaOH ethanol, 0.01 M NaOH ethanol, 0.01 M NaOH water and 0.01 M NaOH methanol for 24 h to remove adsorbed dyes, beads were broken (in 0.1 M NaOH ethanol) or did not completely released dyes (in other solution).

**Fig. 6:**
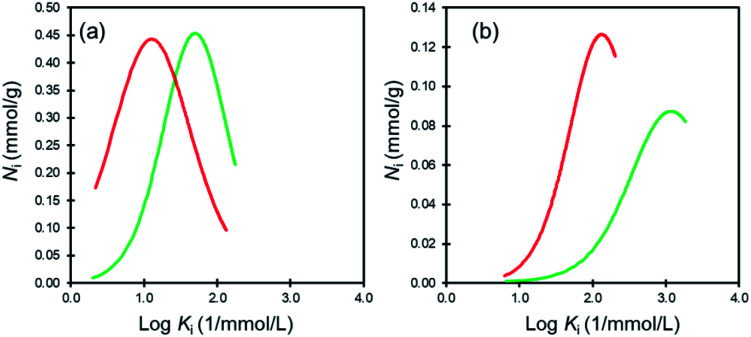
Affinity distributions for (a) Orange II adsorption onto MIL (green line) and MIL-alg (red line) and (b) Rhodamine B adsorption onto MIL (green line) and MIl-alg (red line).

Affinity distribution analysis (see ESI†) was performed to investigate the magnitude of interactions between dye molecules and MIL/MIL-alg.^35,36^ As shown in Fig. 6, unimodal distributions are observed in all cases. In addition, the positions of peak maxima for Rhodamine B are higher than those for Orange II in the presence of both MIL and MIL-alg, indicating that the Rhodamine B has higher adsorption affinities for MIL and MIL-alg. Rhodamine B is a zwitterionic dye (Fig. 2) and both anionic and cationic parts contribute to interactions with the MIL framework. In addition, the larger molecular area of Rhodamine B results in a wider contact area with the MIL. These two factors combine to cause strong Rhodamine B–MIL interactions. Interactions between both dyes and MIL-alg are weaker than those with MIL, and control alg beads show no tendency to adsorb the dyes. Thus, weaker dye adsorption by MIL-alg is likely a consequence of surface coverage by the alginate polymer, which results in a decrease in the MIL contact area.

To gain insights related to interactions between MIL/MIL-alg and the dyes at the molecular level, XPS measurements were conducted. As shown in Fig. S10(a) and (b),† the Fe 2p_3/2_ binding energies of MIL and MIL-alg are slightly shifted to higher energy after dye adsorption, indicating that anionic –SO_3_^−^ and –COO^−^ in Orange II and in Rhodamine B, respectively, interact with unsaturated Fe(iii) sites.

Rates of adsorption are also important factors determining qualities of adsorbents. The results of kinetic experiments reveal that the rates of initial adsorption of Orange II and Rhodamine B on MIL are considerably larger than those on MIL-alg, and that dye adsorption reaches equilibrium within 100 min. For quantitatively analyzing the results, the kinetic data were fitted using several adsorption kinetic models including pseudo first-order (eqn (S3)†), pseudo second-order (eqn (S4)†) and pseudo *n*th-order (eqn (S5)†) models (see ESI and Fig. S11†). For dye adsorption to MIL, the *R*^2^ values for data fit to the pseudo second-order model are slightly higher than those for the pseudo first-order model and similar to those for the pseudo *n*th-order model (Table 2). The parameters *n* for fit to the pseudo *n*th-order model are near 2 for both dyes, indicating that dye adsorption to MIL is best understood using the pseudo second-order model. On the other hand, kinetic data for dye adsorption to MIL-alg are fit best by using the pseudo *n*th-order model (*n* parameters of 3.75 (Orange II) and 3.76 (Rhodamine B)), suggesting that the adsorption mechanism followed by MIL-alg is more complex. Moreover, the rate constants for dye adsorption by MIL are much larger than those for adsorption to MIL-alg, likely because the alginate polymers block diffusion of dyes to the MIL surface. The smaller rate constants for Rhodamine B adsorption as compared to those of Orange II are a consequence of slower diffusion of the larger Rhodamine B in water and the alginate polymer network.

**Table 2:** Kinetic parameters for dye adsorption on MIL and MIL-alg at 30 °C

	Kinetic model	*q*_e_ (mg (g of MIL)^−1^)	*k*_1_ (min^−1^)	*k*_2_ (g (mg min)^−1^)	*k* ((min^−1^) (mg g^−1^)^1−*n*^)	*n*	*R* ^2^
MIL–Orange II	Pseudo-first-order	43.8	1.15 × 10^−1^				0.9970
	Pseudo-second-order	44.9		6.83 × 10^−3^			0.9997
	Pseudo-*n*th-order	44.5			1.43 × 10^−2^	1.74	0.9999
MIL–Rhodamine B	Pseudo-first-order	48.2	1.01 × 10^−1^				0.9958
	Pseudo-second-order	49.6		4.80 × 10^−3^			0.9996
	Pseudo-*n*th-order	49.2			8.60 × 10^−3^	1.81	1.0000
MIL-alg–Orange II	Pseudo-first-order	37.5	2.76 × 10^−2^				0.8541
	Pseudo-second-order	41.5		9.20 × 10^−4^			0.9563
	Pseudo-*n*th-order	55.0			5.74 × 10^−7^	3.75	0.9752
MIL-alg–Rhodamine B	Pseudo-first-order	39.9	8.90 × 10^−3^				0.9866
	Pseudo-second-order	50.8		1.72 × 10^−4^			0.9957
	Pseudo-*n*th-order	74.0			4.70 × 10^−8^	3.76	0.9974

**Fig. 7:**
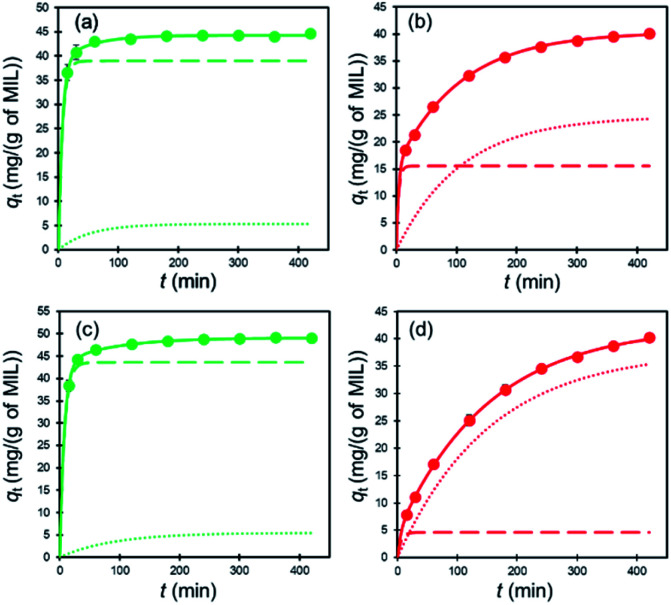
Two-compartment kinetic model analysis for dye adsorption at 30 °C. (a) Orange II adsorption on MIL, (b) Orange II adsorption on MIL-alg, (c) Rhodamine B adsorption on MIL and (d) Rhodamine B adsorption on MIL-alg. The solid lines are fitting curves for the two-compartment kinetic model with fast (dashed lines) and slow (dotted lines) fractions.

The kinetic data were further analyzed using the two-compartment kinetic model (eqn (S6), see ESI†), which assumes that the adsorption pathway is comprised of a fast and a slow step.^37^All data are well fitted by using this model with high *R*^2^ values (see Fig. 7 and Table 3). *F*_fast_ and *F*_slow_ are fractional contributions of the respective fast and slow steps to the rates of adsorption, calculated by using *F*_fast_ = *q*_fast_/*q*_e_ and *F*_slow_ = *q*_slow_/*q*_e_. The results displayed in Table 3 show that *F*_fast_ is much larger than *F*_slow_ for dye adsorption by MIL, while the opposite is the case for MIL-alg where *F*_slow_ is larger than *F*_fast_. Furthermore, the difference between *F*_slow_ and *F*_fast_ for Rhodamine B adsorption is larger than that for adsorption of Orange II in MIL-alg. The results suggest that hinderance to dye adsorption by the presence of alginate polymers causes an increase in *F*_slow_ for MIL-alg and that larger size of Rhodamine B causes an even greater increase in *F*_slow_ for MIL-alg.

**Table 3:** Two compartment kinetic parameters for the dye adsorption on MIL and MIL-alg at 30 °C

	*q*_e_ (mg (g of MIL)^−1^)	Fast step			Slow step			*R* ^2^
		*q*_fast_ (mg (g of MIL)^−1^)	*k*_fast_ (min^−1^)	*F*_fast_	*q*_slow_ (mg (g of MIL)^−1^)	*k*_slow_ (min^−1^)	*F*_slow_	
MIL–Orange II	44.31	38.98	0.156	0.88	5.33	0.019	0.12	0.9919
MIL–Rhodamine B	49.15	43.61	0.131	0.89	5.54	0.011	0.11	0.9996
MIL-alg–Orange II	40.38	15.56	0.271	0.39	24.82	0.009	0.61	0.9995
MIL-alg–Rhodamine B	42.42	4.58	0.177	0.11	37.84	0.007	0.89	0.9998

## Conclusions

In the study described above, a thorough analysis was carried out of Orange II and Rhodamine B dye adsorption on unshaped MIL microcrystals and shaped MIL-alg beads. Observations made by using adsorption modeling and affinity distribution analysis show that shaped MIL-alg beads exhibit slower and weaker adsorption than do unshaped MIL particles as a consequence of the presence of surface blocking alginate polymers. In addition, diffusion of the Rhodamine B dye within alginate polymers is more inhibited than that of Orange II. The combined results lead to the conclusion that the chemical structures and relative amounts of shaping agents require fine degrees of control in fabricating high performance MOF type adsorbents. Future efforts are underway to uncover approaches to improve the dye adsorption properties of MOFs by changing not only the structures of adsorbents and shaping agents but also their ratios.

## Conflicts of interest

There are no conflicts to declare.

## Supplementary Material

RA-011-D1RA03348D-s001
