# Supplementary material for: Influence of carbohydrate polymer shaping on organic dye adsorption by a metal–organic framework in water
Source: RSC Adv. 2021 Jul 6;11(38):23707–13. doi: 10.1039/d1ra03348d (PMC9036605; doi:10.1039/d1ra03348d)
Supplement: RA-011-D1RA03348D-s001 [file RA-011-D1RA03348D-s001.pdf]

Electronic Supplementary Material (ESI) for RSC Advances.
This journal is © The Royal Society of Chemistry 2021

Supporting information

Influence of carbohydrate polymer shaping on organic dye adsorption by a metal-organic framework in water

Yutaro Tanimoto[†] and Shin-ichiro Noro*[,†,‡]

[†]Graduate School of Environmental Science, Hokkaido University, Sapporo 060-0810, Japan
[‡]Faculty of Environmental Earth Science, Hokkaido University, Sapporo 060-0810, Japan

S1. XRF spectra
S2. Elemental analysis
S3. Thermogravimetric curve
S4. N_2 adsorption/desorption isotherm and *t*-plot
S5. SEM-EDX analysis
S6. UV-vis absorption spectra
S7. Comparison of adsorption amounts on various adsorbents for Orange II and Rhodamine B
S8. XPS analysis
S9. Pseudo first-order, pseudo second-order and pseudo *n*th-order model analysis for dye adsorption on MIL and MIL-alg
S10. Adsorption isotherm models, affinity distribution function and adsorption kinetic models
S11. References

S1. XRF spectra

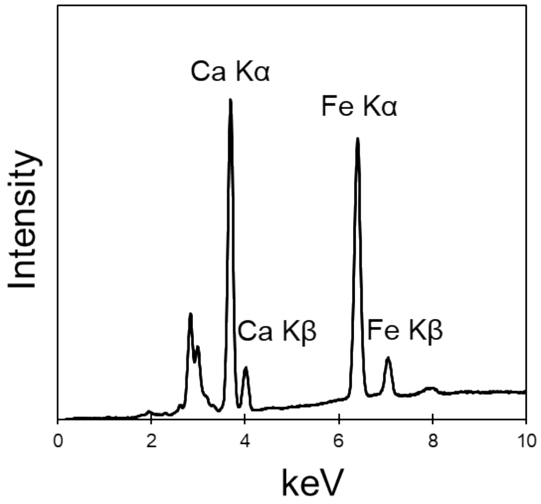

Fig. S1 XRF spectra of dried MIL-alg. The molar ratio of Fe and Ca in dried MIL-alg was calculated from calibration curves, which were obtained using standard samples, iron oxide (Fe_2O_3) and calcium sulfate dihydrate ($CaSO_4 \cdot 2H_2O$), to be Fe:Ca = 1:1.64.

S2. Elemental analysis

Table S1. Observed weight percentage of elements in MIL, dried alg and dried MIL-alg.

Sample	C (%)	H (%)	N (%)	Cl (%)	Others (%)
MIL	33.52	2.16	0.6	-	63.73
MIL-alg	32.72	3.98	-	0	63.34
alg	33.52	4.27	-	-	62.24

From the results of elemental analysis, the formulas of MIL and dried alg were calculated to be $[Fe_3O(H_2O)_2(OH)_{0.7}(NO_3)_{0.3}(1,3,5\text{-benzenetricarboxylate})_2]$ (C 32.54, H 1.62 and N 0.63 %) and $\{[Ca(C_6H_7O_6)_2]\cdot 2H_2O\}$ (C 33.81 and H 4.26 %). It was confirmed that dried MIL-alg is free from $CaCl_2$ and therefore consists of MIL and alg. In addition, nitrogen was not observed in MIL-alg, suggesting that NO_3 anions in MIL are replaced by OH anions during the preparation of MIL-alg beads. Using the composition ratio of Fe:Ca = 1:1.64 (mol) obtained by XRF analysis and the formula of MIL ($[Fe_3O(H_2O)_2(OH)(1,3,5\text{-benzenetricarboxylate})_2]$) and alg ($[Ca(C_6H_7O_6)_2]$), the formula of dried MIL-alg was calculated to be $(MIL)(alg)_{4.92}(H_2O)_{14}$ (C 32.77 and H 3.85 %). High hydrophilicity of alginate beads causes rapid uptake of atmospheric water during handling of dried MIL-alg in the atmosphere.

S3. Thermogravimetric curve

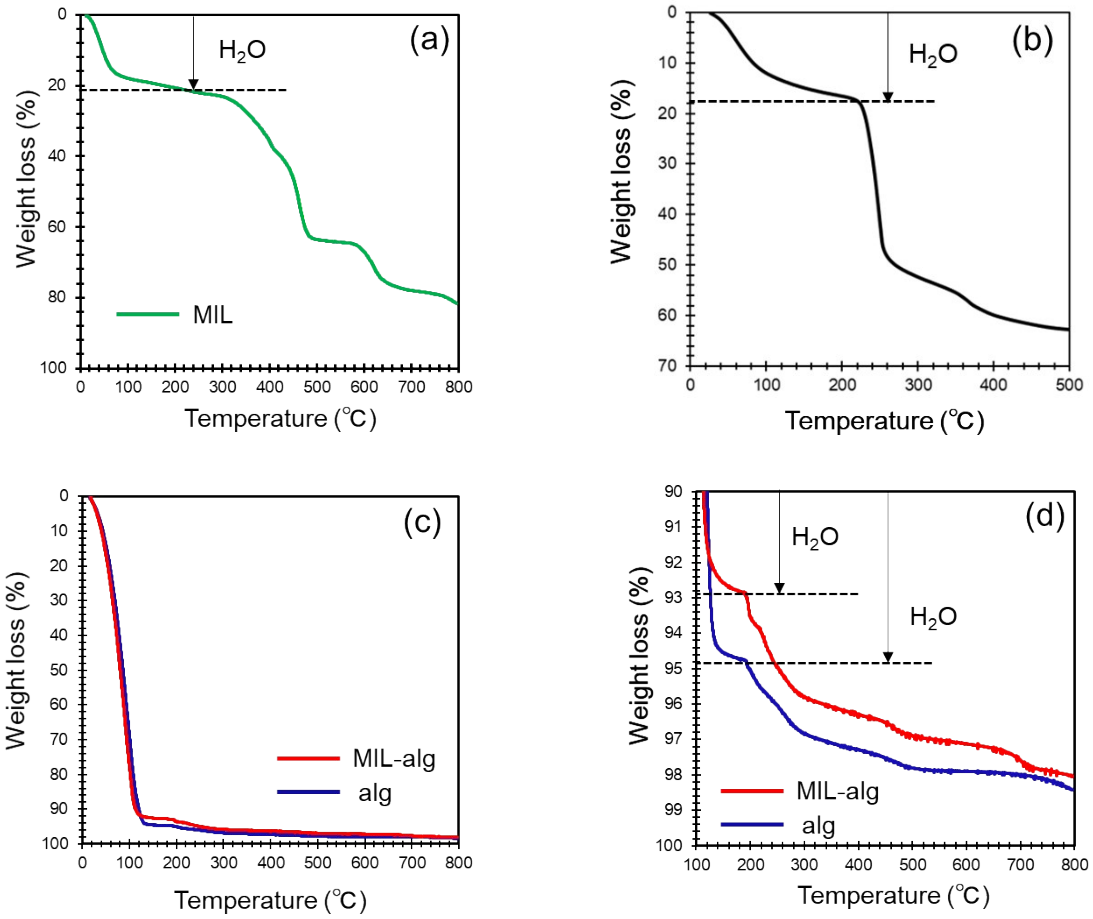

Fig. S2 Thermogravimetric curves of (a) MIL, (b) sodium alginate, (c) one MIL-alg (hydrogel) bead and one alg (hydrogel) bead, and (d) enlarged view of Fig. S2(c). The water content in sodium alginate was calculated from the thermogravimetric curve to be 18.02 %, indicating the formula of $[Na(C_6H_7O_6)]\cdot 2.5H_2O$. The amount of MIL included in one MIL-alg bead was calculated from the thermogravimetric curve and the chemical composition of MIL-alg (MIL:alg = 1:4.92) to be 0.15 mg.

S4. N_2 adsorption/desorption isotherm and *t*-plot

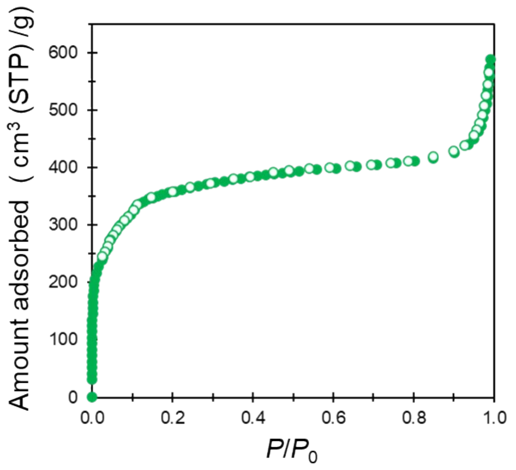

Fig. S3 N_2 adsorption (closed symbol)/desorption (open symbol) isotherm of MIL at 77 K.

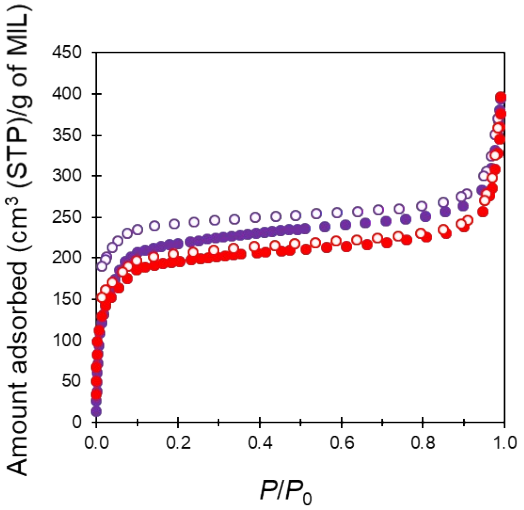

Fig. S4 N_2 adsorption (closed symbols)/desorption (open symbols) isotherms of Orange II adsorbed MIL (red circle) and Rhodamine B adsorbed MIL (purple circle) at 77 K.

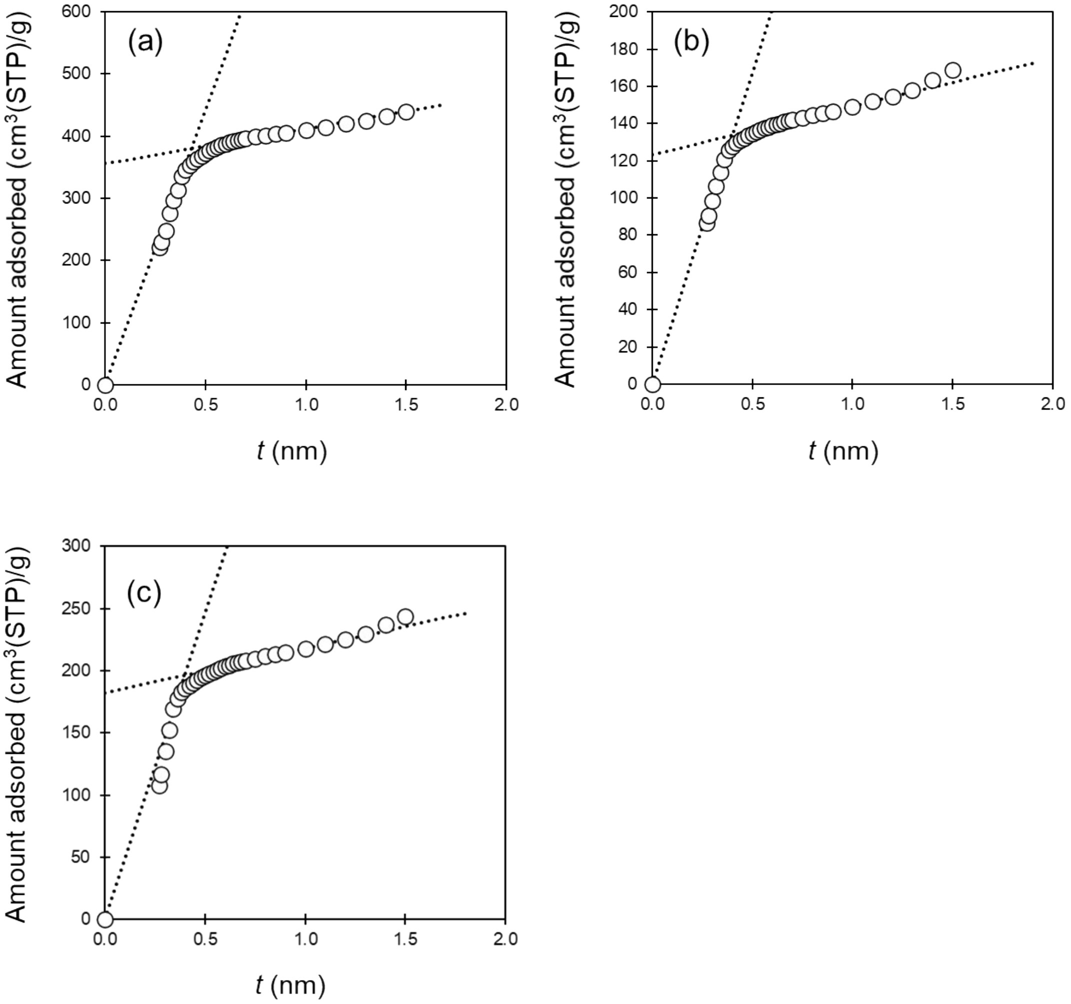

Fig. S5 *t*-plots of N_2 adsorption isotherms for (a) MIL, (b) Orange II adsorbed MIL and (c) Rhodamine B adsorbed MIL.

The theoretical micropore volumes of dye adsorbed MIL assuming that all dye molecules are adsorbed to the outer surface of MIL were calculated using the following equation,

$$V_{theor} = \frac{V_{MIL}}{1 + m_{dye}}$$

where V_{theor} is the theoretical micropore volume of dye adsorbed MIL (cm^3/g), V_{MIL} is the observed micropore volume in MIL (cm^3/g) and m_{dye} is the mass of adsorbed dye (g). The adsorption amounts of dyes were determined in the same procedure described in the main text. V_{exp}, which is the experimental micropore volume (cm^3/g), was calculated from *t*-plots (Fig. S5). The theoretical dye adsorption amounts were calculated using V_{theor} and the dimensions of dye molecules shown in Fig. 5.

Table S2 Micropore volumes, theoretical micropore volumes and theoretical dye adsorption amounts.

	MIL	Orange II adsorbed MIL	Rhodamine B adsorbed MIL
Micropore volume (cm^3/g)	0.55	0.19	0.28
Theoretical micropore volume (cm^3/g)	-	0.37	0.48
Theoretical dye adsorption amount (mg/g)	-	277	100

The theoretical dye adsorption amounts (see Table S2) were lower than the experimental values (424 and 164 mg/g for Orange II and Rhodamine B, respectively) probably because of overestimated molecular dimensions of dyes and removal of adsorbed dyes during the washing process.

The external surface area of MIL was calculated from the *t*-plot. The theoretical monolayer adsorption amounts of dyes on external surface of MIL were calculated using the external surface area and maximum cross-sectional areas of dye molecules estimated from the dimensions in Fig. 5.

Table S3 External surface area of MIL and theoretical monolayer adsorption amounts of dyes on external surface of MIL.

External surface area (m^2/g)	Theoretical monolayer adsorption amount of Orange II (mg/g)	Theoretical monolayer adsorption amount of Rhodamine B (mg/g)
73.57	29.15	23.90

S5. SEM-EDX analysis

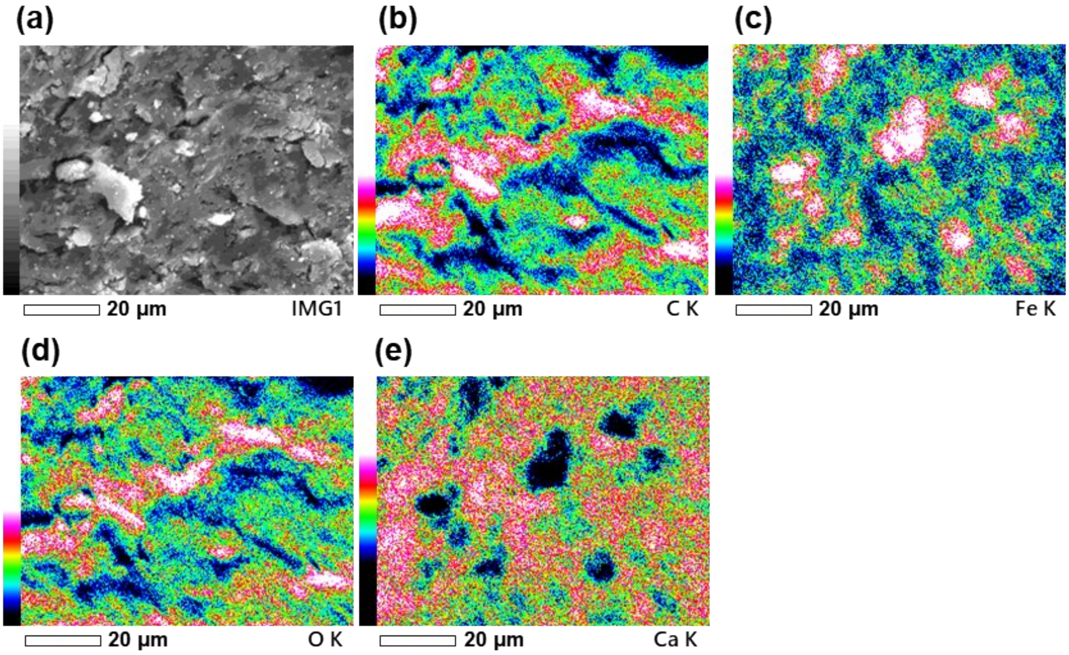

Fig. S6 (a) SEM image and (b-e) EDX maps ((b) C, (c) Fe, (d) O and (e) Ca) of dried MIL-alg.

Table S4. Weight percentage data obtained from the EDX analysis (Fig. S6).

	C (%)	O (%)	Ca (%)	Fe (%)
MIL-alg	33.89	37.32	16.48	12.3

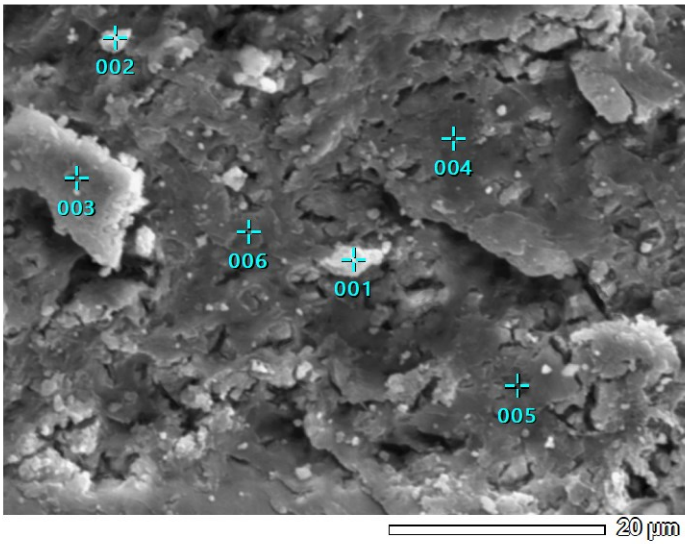

Fig. S7 SEM-EDX point analysis of dried MIL-alg.

Table S5. Weight percentage data obtained from the point analysis (Fig. S7).

Point	C (%)	O (%)	Ca (%)	Fe (%)
1	42.92	40.08	13.07	3.94
2	40.33	38.57	7.60	13.51
3	38.92	34.65	20.14	6.29
4	33.06	35.61	23.74	7.59
5	30.74	32.09	27.51	9.65
6	32.63	36.79	24.79	5.80

S6. UV-vis absorption spectra

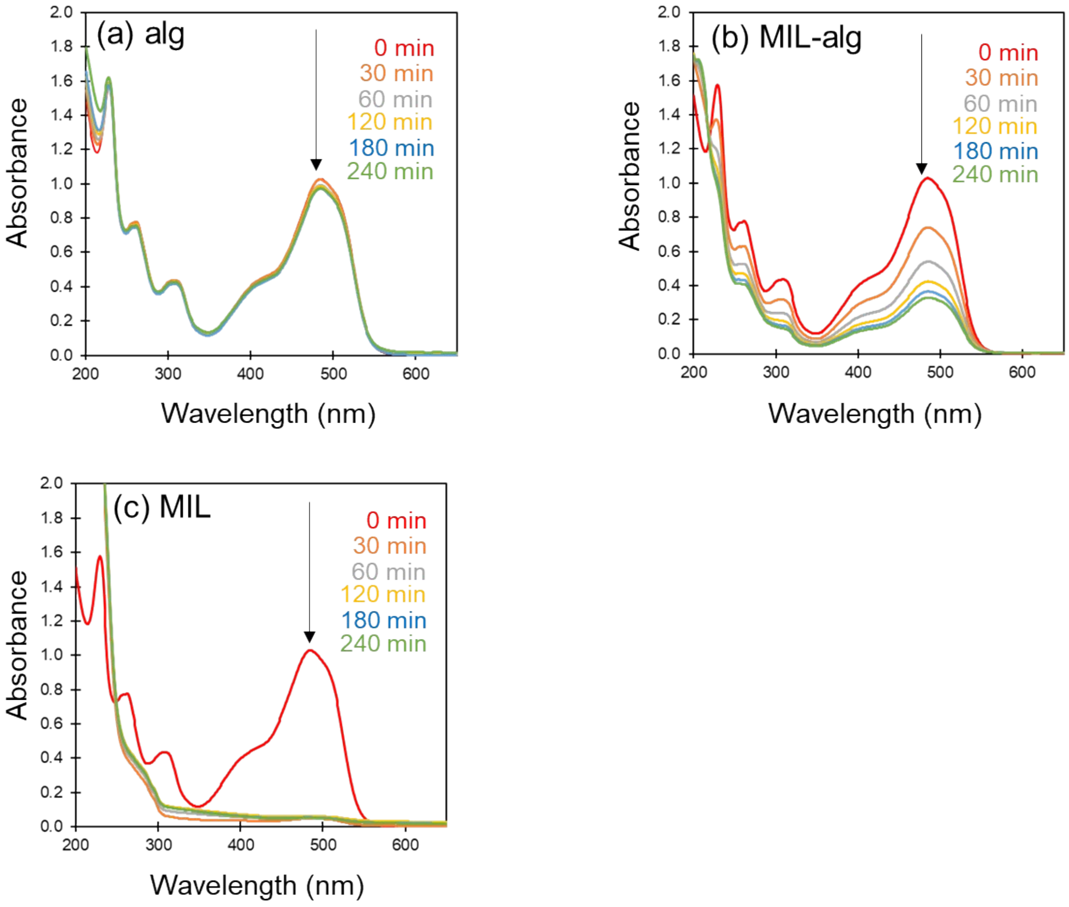

Fig. S8 UV-vis spectral change of aqueous Orange II solution (15 mg/L) in the presence of (a) alg (10 beads), (b) MIL-alg (10 beads) and (c) MIL (10 mg).

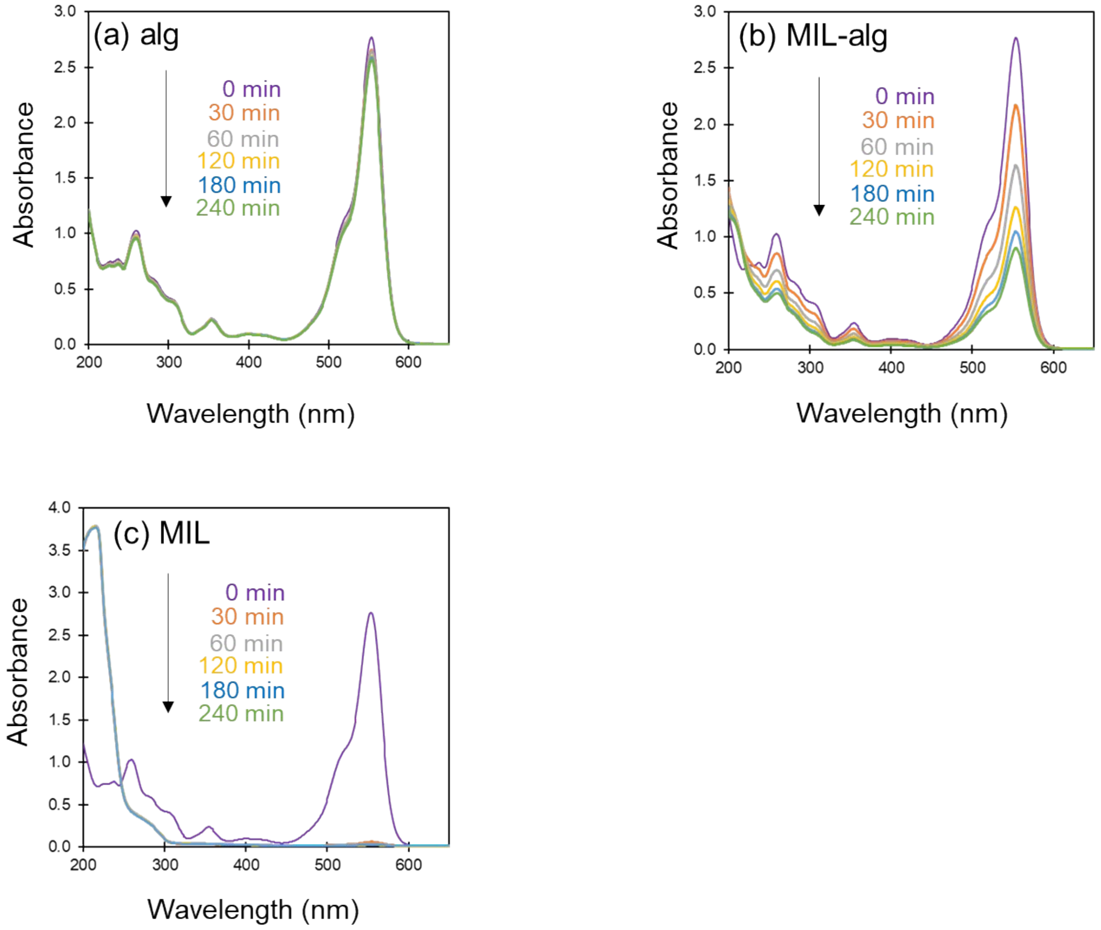

Fig. S9 UV-vis spectral change of aqueous Rhodamine B solution (15 mg /L) in the presence of (a) alg (10 beads), (b) MIL-alg (10 beads) and (c) MIL (10 mg).

S7. Comparison of adsorption amounts on various adsorbents for Orange II and Rhodamine B

Table S6. Orange II and Rhodamine B adsorption amounts on previously reported adsorbents.

Dye	Adsorbent	Surface area (m^2/g)	Adsorption amount (mg/g)	Temperature (°C)	pH	Reference
Orange II	$[Cu(4,4'\text{-bipyridine})Cl_2]_n$	5.8	921	27	—	(S1)
	$[Cu(4,4'\text{-bipyridine})(SO_4)]_n$	5.3	3308	27	—	(S1)
	Hexadecyltrimethylammonium bromide-coated zeolite	—	38.96	30	1.0	(S2)
	Polyaniline/bentonite nanocomposite	7.5	105.9	25	2.0	(S3)
	Zirconium-based chitosan microcomposite	6.24	926	30	2.0	(S4)
	Cationic surfactant modified biochar pyrolyzed from cornstalk	—	29.1	35	7.0	(S5)
	Kapok fiber oriented polyaniline	21.8	188.7	30	7.0	(S6)
	Aluminum oxide nanoparticle	82.91	97.6	30	—	(S7)
	MIL-100(Fe)	2037	410	25	3.0	(S8)
	MIL	1286	424	30	6.9	This work
	MIL-alg	—	541	30	6.9	This work
Rhodamine B	Waste of seeds of *Aleurites Moluccana*	—	82	25	6.0	(S9)
	Treated rice husk-based activated carbon	892	478.5	30	—	(S10)
	Graphite oxide	71.5	578	30	—	(S11)
	$MnFe_2O_4$ nanoparticles	53.99	9.3	25	10.5	(S12)
	MIL-68(Al)	976	1111.1	—	—	(S13)
	Modified zeolite	—	4.41	30	7.0	(S14)
	Nano scale MIL-100(Fe)	1.24	73	35	—	(S15)
	Hierarchical SnS_2 nanostructure	48.6	200	25	—	(S16)
	MIL	1286	164	30	5.5	This work
	MIL-alg	—	161	30	5.5	This work

S8. XPS analysis

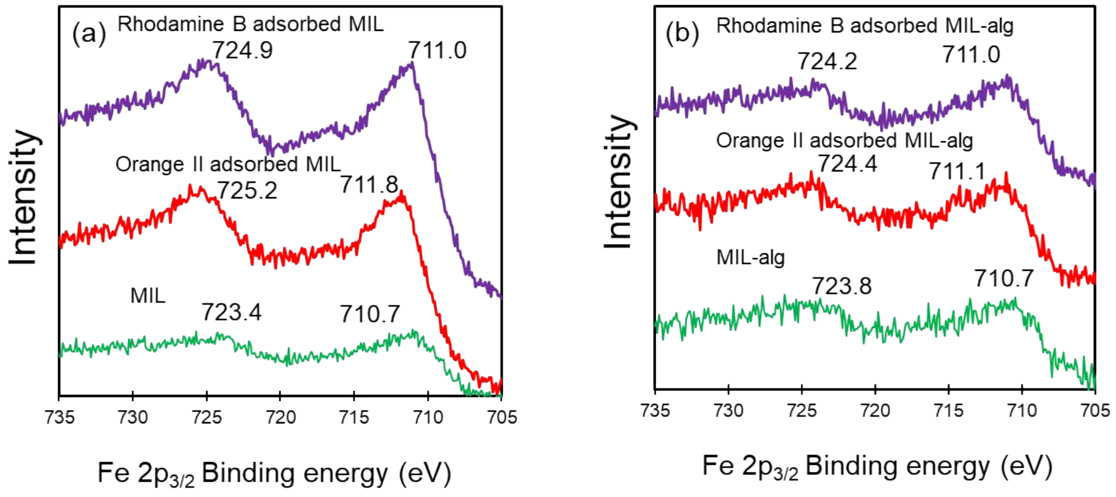

Fig. S10 XPS spectra of (a) Fe $2p_{3/2}$ in MIL, Orange II adsorbed MIL and Rhodamine B adsorbed MIL and (b) Fe $2p_{3/2}$ in MIL-alg, Orange II adsorbed MIL-alg and Rhodamine B adsorbed MIL-alg.

S9. Pseudo first-order, pseudo second-order and pseudo *n*th-order model analysis for dye adsorption on MIL and MIL-alg

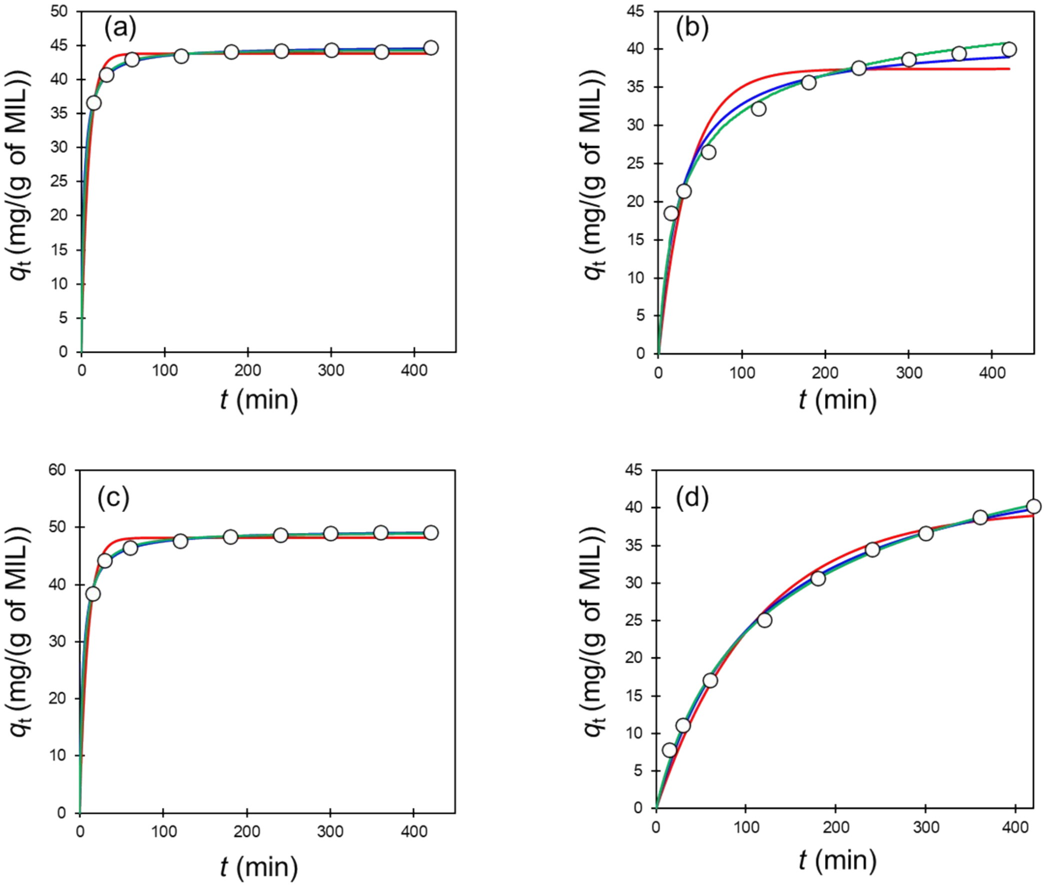

Fig. S11 Pseudo first-order, pseudo second-order and pseudo *n*th-order model analysis for dye adsorption at 30 ºC. (a) Orange II adsorption on MIL, (b) Orange II adsorption on MIL-alg, (c) Rhodamine B adsorption on MIL and (d) Rhodamine B adsorption on MIL-alg. The red, blue and green lines represent fitting curves for the pseudo first-order, pseudo second-order and pseudo *n*th-order models, respectively.

S10. Adsorption isotherm models, affinity distribution function and adsorption kinetic models

The adsorption isotherms were fitting using the Langmuir-Freundlich (LF) model (equation S1),[S17,S18]

$$q_e = \frac{q_m a C_e^n}{1 + a C_e^n} \qquad \text{(S1)}$$

where q_e is the adsorption amount (mg/g), q_m is the theoretical maximum adsorption amount (mg/g), a is the affinity constant and n is the heterogeneity parameter with the range from 0 to 1 and C_e is the equilibrium concentration (mg/L).

The affinity distribution analysis based on the LF model was performed using the equation S2,[S17,S18]

$$N_i = q_m a n (1/K_i)^n \times$$

$$\frac{(1 + 2a(1/K_i)^n + a^2(1/K_i)^{2n} + 4a(1/K_i)^n n^2 - a^2(1/K_i)^{2n} n^2 - n^2}{(1 + a(1/K_i)^n)^4} \qquad \text{(S2)}$$

where N_i is the number of adsorption sites and K_i is the association constant. K_i has the range between $K_{max} = 1/C_{min}$ and $K_{min} = 1/C_{max}$, in which C_{min} and C_{max} are the maximum and minimum equilibrium concentrations, respectively.

The adsorption kinetic analysis was performed using the following pseudo first-order (eq S3), pseudo second-order (equation S4) and pseudo nth-order (equation S5) models,[S19,S20]

$$q_t = q_e[1 - \exp(-k_1 t)] \qquad \text{(S3)}$$

$$q_t = \frac{k_2 q_e^2 t}{1 + k_2 q_e t} \qquad \text{(S4)}$$

$$q_t = q_e - [(n-1)kt + q^{(1-n)}]^{1/(1-n)} \qquad \text{(S5)}$$

where q_t is the adsorption amount at time t (mg/g), q_e is the equilibrium adsorption amount (mg/g), k_1, k_2 and k are the kinetic rate constants and n is the reaction order.

The adsorption kinetic data were also analyzed using the following two-compartment kinetic model (equation S6),[S21]

$$q_e = q_{fast}[1 - exp(-k_{fast}t)] + q_{slow}[1 - exp(-k_{slow}t)] \quad \text{(S6)}$$

where q_{fast} and q_{slow} are the adsorption amounts of dye per gram of MIL (mg/g) at time *t* (min) of fast and slow adsorption, respectively, and k_{fast} and k_{slow} are the adsorption rate constants (1/min).

S11. References

S1 L. Xiao, Y. Xiong, Z. Wen, S. Tian, *RSC Adv.*, 2015, **5**, 61593-61600.

S2 X. Jin, B. Yu, Z. Chen, J. M. Arocena, R. W. Thring, *J. Colloid Interface Sci.*, 2014, **435**, 15-20.

S3 W. Li, Q. Lin, M. Gao, H. Ma, *Water Sci. Technol.*, 2017, **76**, 337-354.

S4 L. Zhang, L. Chen, X. Liu, W. Zhang, *RSC Adv.*, 2015, **5**, 93840-93849.

S5 X. Mi, G. Li, W. Zhu, L. Liu, *J. Chem.*, 2016, **4**, 1-7.

S6 Y. Zheng, Y. Liu, A. Wang, *Ind. Eng. Chem. Res.*, 2012, **51**, 10079-10087.

S7 E. Khosla, S. Kaur, P. N. Dave, *J. Eng.*, 2013, **1**, 1-8.

S8 F. Tsai, Y. Xia, N. Ma, J. Shi, T. Jiang, T. Chiang, Z. Zhang, W. Tsen, *Desalination Water Treat.*, 2016, **57**, 3218–3226.

S9 D. L. Postai, C. A. Demarchi, F. Zanatta, D. C. C. Melo, C. A. Rodrigues, *ALEX. ENG. J.*, 2016, **55**, 1713-1723.

S10 L. Ding, B. Zou, W. Gao, Q. Liu, Z. Wang, Y. Guo, X. Wang, Y. Liu, *Colloids Surf. A Physicochem. Eng. Asp.*, 2014, **446**, 1-7.

S11 M. Yin, Y. Pan, C. Pan, *Micro & Nano Letters*, 2019, **14**, 1192-1197.

S12 U. Lamdab, K. Wetchakun, W. Kagwansupamonkon, N. Wetchakun, *RSC Adv.*, 2018, **8**, 6709-6718.

S13 M. S. Tehrani, R. Zare-Dorabei, *RSC Adv.*, 2016, **6**, 27416-27425.

S14 W. Y. Cheng, N. Li, Y. Z. pan, L. H. Jin, *Mod. Appl. Sci.*, 2016, **5**, 67-76.

S15 S. Duan, J. Li, X. Liu, Y. Wang, S. Zeng, D. Shao, T. Hayat, *ACS Sustainable Chem. Eng.*, 2016, **4**, 3368–3378.

S16 S. Wang, B. Yang, Y. Liu, *J. Colloid Interface Sci.*, 2017, **507**, 225-233.

S17 R. J. Umpleby II, S. C. Baxter, A. M. Rampey, G. T. Rushton, Y. Chen, K. D. Shimizu, *J. Chromatogr. B*, 2004, **804**, 141–149.

S18 R. J. Umpleby II, S. C. Baxter, Y.; Chen, R. N. Shah, K. D. Shimizu, *ACS Anal. Chem.*, 2001**,** **73**, 4584–4591.

S19 S. Asadi, S. Eris, S. Azizian, *ACS Omega*, 2018, **3**, 15140–15148.

S20 A. Özer, *J. Hazard. Mater.* 2007, **141**, 753–761.

S21 Choi, S. R. Al-Abed, *J. Hazard. Mater.*, 2009, **165**, 860–866.
